# Large-scale clinical validation of biomarkers for pancreatic cancer using a mass spectrometry-based proteomics approach

**DOI:** 10.18632/oncotarget.17463

**Published:** 2017-04-27

**Authors:** Jisook Park, Eunjung Lee, Kyoung-Jin Park, Hyung-Doo Park, Jong-Won Kim, Hye In Woo, Kwang Hyuck Lee, Kyu-Taek Lee, Jong Kyun Lee, Joon-Oh Park, Young Suk Park, Jin Seok Heo, Seong Ho Choi, Dong Wook Choi, Kee-Taek Jang, Soo-Youn Lee

**Affiliations:** ^1^ Samsung Biomedical Research Institute, Samsung Medical Center, Sungkyunkwan University School of Medicine, Seoul, Korea; ^2^ Division of Genetics and Genomics, Boston Children's Hospital and Harvard Medical School, Boston, MA, United States; ^3^ Department of Laboratory Medicine and Genetics, Samsung Medical Center, Sungkyunkwan University School of Medicine, Seoul, Korea; ^4^ Department of Laboratory Medicine, Samsung Changwon Hospital, Sungkyunkwan University School of Medicine, Changwon, Korea; ^5^ Division of Gastroenterology, Department of Medicine, Samsung Medical Center, Sungkyunkwan University School of Medicine, Seoul, Korea; ^6^ Division of Hematology-Oncology, Department of Medicine, Samsung Medical Center, Sungkyunkwan University School of Medicine, Seoul, Korea; ^7^ Department of Surgery, Samsung Medical Center, Sungkyunkwan University School of Medicine, Seoul, Korea; ^8^ Department of Pathology and Translational Genomics, Samsung Medical Center, Sungkyunkwan University School of Medicine, Seoul, Korea; ^9^ Department of Clinical Pharmacology and Therapeutics, Samsung Medical Center, Sungkyunkwan University School of Medicine, Seoul, Korea

**Keywords:** pancreatic cancer, biomarker, validation, proteomics, mass spectrometry

## Abstract

We performed an integrated analysis of proteomic and transcriptomic datasets to develop potential diagnostic markers for early pancreatic cancer. In the discovery phase, a multiple reaction monitoring assay of 90 proteins identified by either gene expression analysis or global serum proteome profiling was established and applied to 182 clinical specimens. Nine proteins (*P* < 0.05) were selected for the independent validation phase and quantified using stable isotope dilution-multiple reaction monitoring-mass spectrometry in 456 specimens. Of these proteins, four proteins (apolipoprotein A-IV, apolipoprotein CIII, insulin-like growth factor binding protein 2 and tissue inhibitor of metalloproteinase 1) were significantly altered in pancreatic cancer in both the discovery and validation phase (*P* < 0.01). Moreover, a panel including carbohydrate antigen 19-9, apolipoprotein A-IV and tissue inhibitor of metalloproteinase 1 showed better performance for distinguishing early pancreatic cancer from pancreatitis (Area under the curve = 0.934, 86% sensitivity at fixed 90% specificity) than carbohydrate antigen 19-9 alone (71% sensitivity).

Overall, we present the panel of robust biomarkers for early pancreatic cancer diagnosis through bioinformatics analysis that combined transcriptomic and proteomic data as well as rigorous validation on a large number of independent clinical samples.

## INTRODUCTION

Pancreatic cancer (PC) is the fifth most common cause of cancer-related mortality worldwide. Only 10 to 20% of patients are eligible for surgical resection with curative intent [1, 2]. More than half of patients are diagnosed after distant metastasis, for which 5-year the survival rate is only 2% [2]. Operative resection, the only potentially curative treatment for PC, is not applicable in cases of advanced or metastatic PC. Survival rates are stage-dependent; the 5-year survival rate of PC confined to the pancreas is approximately 26%, compared with only 7% for all stages combined [3].

Although screening methods using a combination of imaging, invasive, and biochemical studies have been evaluated for the early detection of PC in high-risk individuals, these approaches showed limited diagnostic performance [4–6]. There is currently a lack of low-cost noninvasive methods for the early detection of PC [7]. Carbohydrate antigen 19-9 (CA 19-9) has been used for the evaluation of operability and recurrence and for monitoring PC, usually in combination with clinical findings and imaging studies [8]. CA 19-9 is not recommended for use in screening for PC because of its limited sensitivity and specificity of 70–92% and 68–92% respectively for the diagnosis of PC [8–13]. In particular, it is unsuitable for detection of early-stage PC because of its low sensitivity (~50%) [9, 10].

High-throughput technology for proteomics (e.g., development of instruments, separation techniques for crude samples) that can simultaneously handle a tremendous number of proteins has recently been introduced. Shotgun proteomics leads to the identification of proteins that are potentially associated with cancer conditions. In PC, proteomic profiling in biological tissues [12–14] and fluids [4, 7, 15–17] has been applied to discover novel biomarkers using surface-enhanced laser desorption/ionization-mass spectrometry (SELDI-MS), matrix-assisted laser desorption/ionization-mass spectrometry (MALDI- TOF-MS), liquid chromatography tandem mass spectrometry (LC-MS/MS) and two-dimensional gel electrophoresis (2DE).

For example, alterations of several proteins (e.g., annexin A4, cyclophilin A, cathepsin D, galectin-1, 14-3-3ζ, α-enolase, peroxiredoxin I, TM2, and S100A8) were reported in PC compared with normal and pancreatitis tissues by a 2DE and MS approach [13]. Using isotope-coded affinity tags (ICAT) technology, Bronner et al. identified differentially expressed proteins (e.g., annexin A2 and insulin-like growth factor-binding protein 2 [IGFBP2]) in PC tissue compared to chronic pancreatitis. They reported that annexin A2 and IGFBP2 were exclusively overexpressed in PC whereas cathepsin D, integrin beta1, and plasminogen were overexpressed in both pancreatic cancer and chronic pancreatitis [14]. The secretome from PC-derived cells was analyzed and 145 differentially expressed proteins were identified using stable isotope labeling with amino acids in cell culture (SILAC) [16]. Five proteins (cyclin I, Rab GDP dissociation inhibitor beta [GDI2], alpha-1 antitrypsin precursor, haptoglobin precursor, and serotransferrin precursor) were suggested as biomarker candidates using 2DE and MS [7]. Apolipoprotein-AII (APOAII) isoforms (especially APOAII-2) have been suggested to be potential biomarker surrogates for pancreatic cancer and their clinical usefulness has been evaluated via multi-institutional validation [17]. More recently, Honda et al. developed the ELISA for measurement of APOAII-2 and performed multi-institutional validation of the usefulness of APOAII-2 as a pancreatic cancer biomarker [18].

Although intensive biomarker investigations have previously been performed, there are major challenges to their validation for clinical use, such as methods for developing, evaluating and implementing the biomarkers and for collection of clinical samples [5]. The use of ELISA, a commonly used antibody-based approach, for candidate biomarkers is limited by the need for an appropriate antibody and the long period required for assay development [6, 19]. Quantitative methodologies that can be applied in a rapid high-throughput fashion are needed for the prioritization of a large number of candidates, allowing for the extraction of clinically relevant biomarkers in both the discovery and validation phase. Therefore, we established a multiple reaction monitoring (MRM)-based quantitative assay with stable isotope-labeled standard peptides [20] and then applied the assay to a large number of clinical specimens for the clinical validation of PC biomarker candidates.

## RESULTS

### Biomarker development pipeline

**Figure 1:**
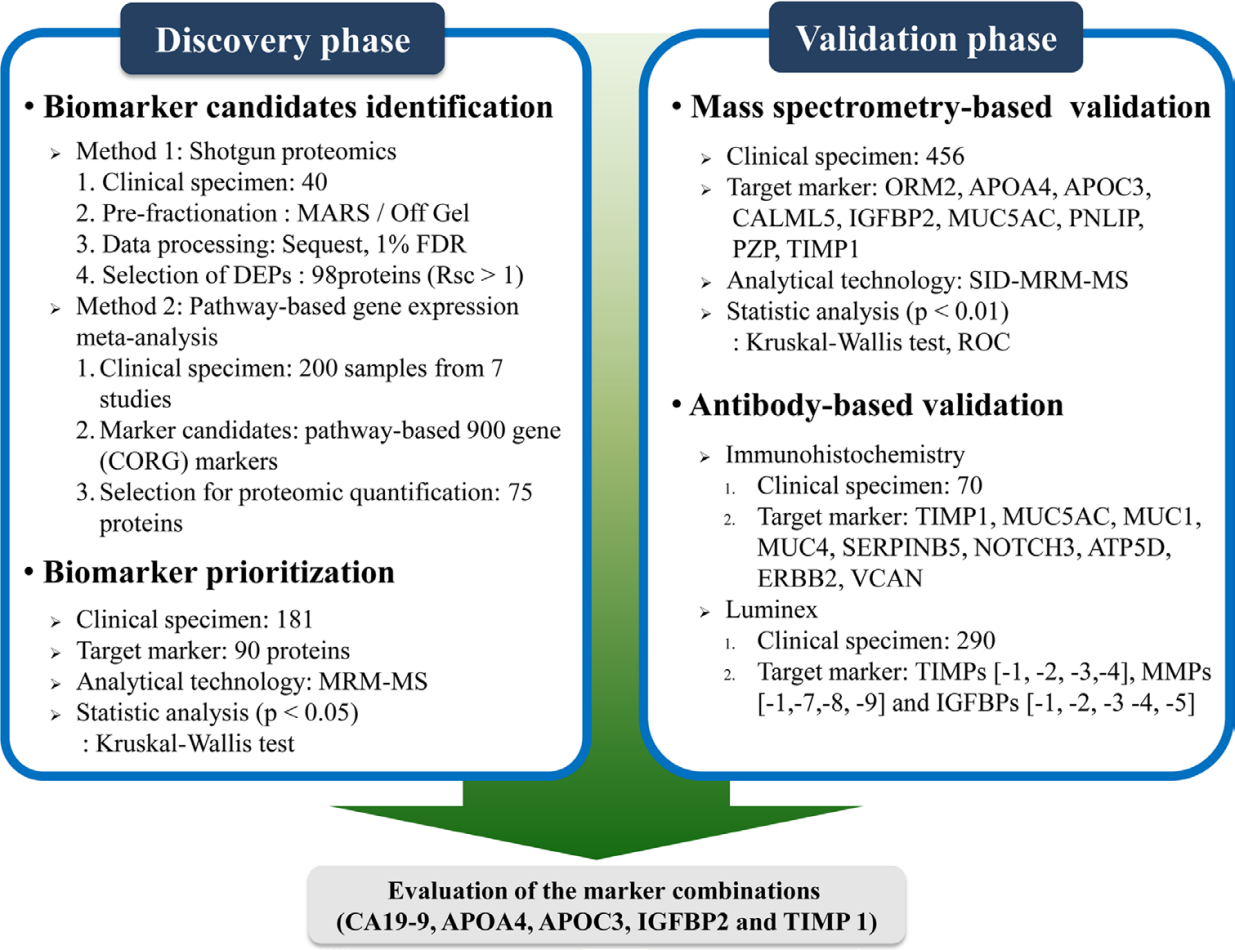
Brief workflow of pancreatic cancer associated-biomarker mining In the biomarker discovery phase, a shotgun proteomics approach and pathway-based gene expression meta-analysis were performed to identify potential biomarkers for early pancreatic cancer diagnosis. Ninety MRM assays were established and performed on 182 clinical samples. These proteins were prioritized according to statistical evidence (*P* values ≤ 0.05), and nine proteins were chosen for the biomarker validation phase. The serum levels of these proteins were determined on 456 clinical specimens using SID-MRM-MS. Additionally, immunohistochemistry staining of nine proteins was performed on 70 patient-derived pancreatic tissues. Levels of TIMP-1, -2, -3 and -4, MMP-1,-7,-8 and -9, and IGFBP-1, -2, -3 -4 and -5 were determined for 290 clinical specimens using Luminex. Based on the results, APOA4, APOC3, IGFBP2 and TIMP1 were selected for biomarker panel generation, in combination with CA 19-9.

The brief workflow of PC associated-biomarker development is shown in Figure 1. For the discovery of biomarkers, shotgun proteomics was performed with three samples of pooled sera (10 healthy controls, 10pancreatitis and 20 PCs) and targeted proteomics analysis was carried out with serum specimens from 182 individuals (42 early [stage I/II] PCs, 74 advanced [stage III/IV] PCs, 31 cases of pancreatitis, and 35 healthy controls). Then, we created an independent validation set consisting of 456 specimens (292 PCs, 71 cases of pancreatitis, and 94 healthy subjects) that was used to validate the markers of interest (Table 1).

**Table 1:** Baseline characteristics

	Discovery set (*N* = 182)			Validation set (*N* = 456)		
	Healthy subjects	Pancreatitis	Pancreatic cancer	Healthy subjects	Pancreatitis	Pancreatic cancer
*N*	35	31	116	94	70	292
Sex* (Male/Female)	20/15	11/20	63/53	58/36	55/15	176/116
Age*, years						
< 60	23	14	57	40	46	123
60–70	11	11	38	36	12	101
> 70	1	6	21	18	12	68
BMI*, kg/m^2^						
≤ 30	35	26	113	94	70	287
> 30	0	0	3	0	0	5
Unknown		5				
Smoking status*						
No	19	10	69	50	35	136
Yes	12	12	45	42	31	137
Unknown	4	9	2	2	4	19
Diagnosed with diabetes						
Yes	1	15	29	2	30	104
No	34	16	87	92	40	180
Unknown						8
Cancer histological type						
Ductal adenocarcinoma			103			280
Neuroendocrine carcinoma			9			5
Others			4			7
TNM classification						
Stage I/II			0/42			4/57
Stage III/IV			25/49			26/204

NOTE: *P*-values were calculated using Pearson's chi-square test to assess differences in age, sex, BMI, smoking status and diabetes among groups. *P > 0.05 on Pearson's chi-square test.

We applied an integrated analysis of proteomic and transcriptomic data in order to identify potential biomarkers for PC diagnosis. In the discovery phase, 117 proteins and 98 proteins were identified using pathway-based gene expression meta-analysis and shotgun proteomics, respectively (Supplementary Table 1). MRM-based protein assays were developed for 90 proteins and used to measure levels of these proteins in 182 clinical samples. The serum concentrations of the isolated 90 proteins were prioritized according to statistical evidence.

Nine proteins that exhibited differential expression in PC were subjected to the validation phase on 456 clinical specimens using SID-MRM-MS. For additional antibody-based validation, IHC staining of the nine proteins was additionally performed on 70 samples of patient tissues dissected from normal ducts, pancreatitis and PC lesions. Serum levels of 13 proteins (tissue inhibitor of metalloproteinase [TIMP] -1, -2, -3 and -4, [MMP]-1,-7, -8 and -9, and insulin-like growth factor binding protein [IGFBP] -1, -2, -3 -4 and -5) were measured using the Luminex assay in 102 controls and 118 PCs.

Four proteins (apolipoprotein A-IV [APOA4], apolipoprotein CIII [APOC3], insulin-like growth factor binding protein 2 IGFBP2] and tissue inhibitor of metalloproteinase 1 [TIMP1]) were finally chosen as potential biomarkers. We developed a panel of markers consisting of these proteins combined with CA 19-9 and evaluated its diagnostic performance to identify early PC.

### Biomarker candidate identification

We performed a pathway-based meta-analysis of seven previous studies comprised of 200 microarray experiments on PC, chronic pancreatitis, and normal pancreas samples. This analysis identified a total of 581 pathway candidates and 980 CORG marker candidates prioritized by their reproducibility in predicting disease phenotypes across the studies. Among the CORG marker candidates, we excluded proteins associated with non-specific systemic inflammation except for those that were differentially expressed in chronic pancreatitis. After consideration of DNA copy number changes and cellular localization of the remaining CORG marker candidates, we selected 117 proteins for further proteomic quantitative analysis.

A total of 16,714 MS/MS spectra corresponding to 334 proteins were identified in the discovery phase with a 1% false discovery rate (FDR) by LC-ESI-MS/MS. MS/MS spectra were obtained in each experimental group: 3,188 of 181 proteins in advanced PC; 4,522 of 191 proteins in early PC; 3,847 of 233 proteins in pancreatitis; and 5,157 of 206 proteins in healthy controls. To identify potential biomarkers, we performed semi-quantification by spectral counts, which represent the abundance of each protein. To avoid experimental bias and variation we used the ratio of spectral counts (Rsc-value) [21], which is the log_2_ ratio of protein abundance between groups. We chose 98 proteins with Rsc > 1, indicating 2-fold changes in comparisons between groups.

### Biomarker prioritization based on MRM assay

We developed a MRM assay of 90 among the 215 proteins derived from the discovery phase using the following criteria: (i) At least three MRM transitions per peptide should be extracted at the same retention time with S/N > 8, and (ii) coefficient of variation (CV) < 20% (data not shown).

MRM assay of the 90 proteins that satisfied the above criteria was carried out on 182 individuals. Nine markers that were altered in PCs compared with pancreatitis or healthy controls using Kruskal-Wallis test (*P* < 0.05) were finally selected. Five proteins (ORM2, APOA4, APOC3, CALML5 and PZP) were identified through shotgun proteomics and 4 proteins (IGFBP2, MUC5AC, PNLIP and TIMP1) were identified from pathway-based gene expression meta-analysis followed by MRM-MS (Table 2).

**Table 2:** MRM results and significance tests for selected marker proteins

Markers	Discovery set (*N* = 182)				Validation set (*N* = 456)			
	Median (95% CI)^§^			*P*-value*	Median (95% CI), Amol/uL			*P*-value*
	Healthy subjects (*N* = 35)	Pancreatitis (*N* = 31)	Pancreatic cancer (*N* = 116)		Healthy subjects (*N* = 94)	Pancreatitis (*N* = 70)	Pancreatic cancer (*N* = 292)	
ORM2	827 (510–1145)	1291 (671–1911)	1696 (1405–1987)	< 0.001	1405 (482–2468)	2025 (1094–3797)	2294 (1474–3171)	0.154
APOA4	49 (24–73)	49 (33–65)	32 (27–38)	0.004	7436 (6090–9154)	9565 (7973–14356)	3925 (3470–4690)	< 0.001
APOC3	465 (361–569)	376 (286–466)	265 (232–299)	< 0.001	1536 (783–2607)	3310 (1686–4761)	1094 (913–1622)	0.006
CALML5	16 (116–21)	13 (10–16)	23 (21–26)	< 0.001	0 (0–80)	90 (43–150)	90.0 (52–130)	0.02
IGFBP2	14 (7–36)	6 (2–66)	5 (3–11)	< 0.001	3648 (381–11366)	19465 (3382–27303)	217 (160–301)	< 0.001
MUC5AC	5 (3–9)	3 (1–6)	6 (1–22)	0.003	5 (0–40)	58 (23–98)	10 (0–30.0)	0.023
PNLIP	9 (4–20)	12 (7–80)	23 (4–64)	< 0.001	963 (313–1500)	1770 (980–2417)	723 (287–1080)	0.159
PZP	265 (193–338)	281 (186–377)	383 (325–441)	0.002	74400 (68429–81531)	75773 (73301–82353)	71144 (69177–74952)	0.427
TIMP1	30 (22–38)	17 (11–22)	12 (11–14)	< 0.001	185 (50–260)	180 (130–247)	230 (148–300)	0.003

NOTE: ^§^Beta galactosidase peptide (VDEDQPFPAVPK) was used for the relative quantification of target proteins. Each protein level was normalized against 50 fmol/μL of beta galactosidase. Abbreviations: CA 19-9, carbohydrate antigen 19-9; ORM2, alpha-1-acid glycoprotein 2; APOA4, apolipoprotein A-IV; APOC3, apolipoprotein CIII; CALML5, calmodulin-like protein 5; IGFBP2, insulin-like growth factor binding protein 2; MUC5AC, mucin 5AC glycoprotein; PNLIP, pancreatic triacylglycerol lipase; PZP, pregnancy zone protein; TIMP1, tissue inhibitor of metalloproteinase 1; CI, confidence interval

### Biomarker validation with SID-MRM-MS

**Figure 2:**
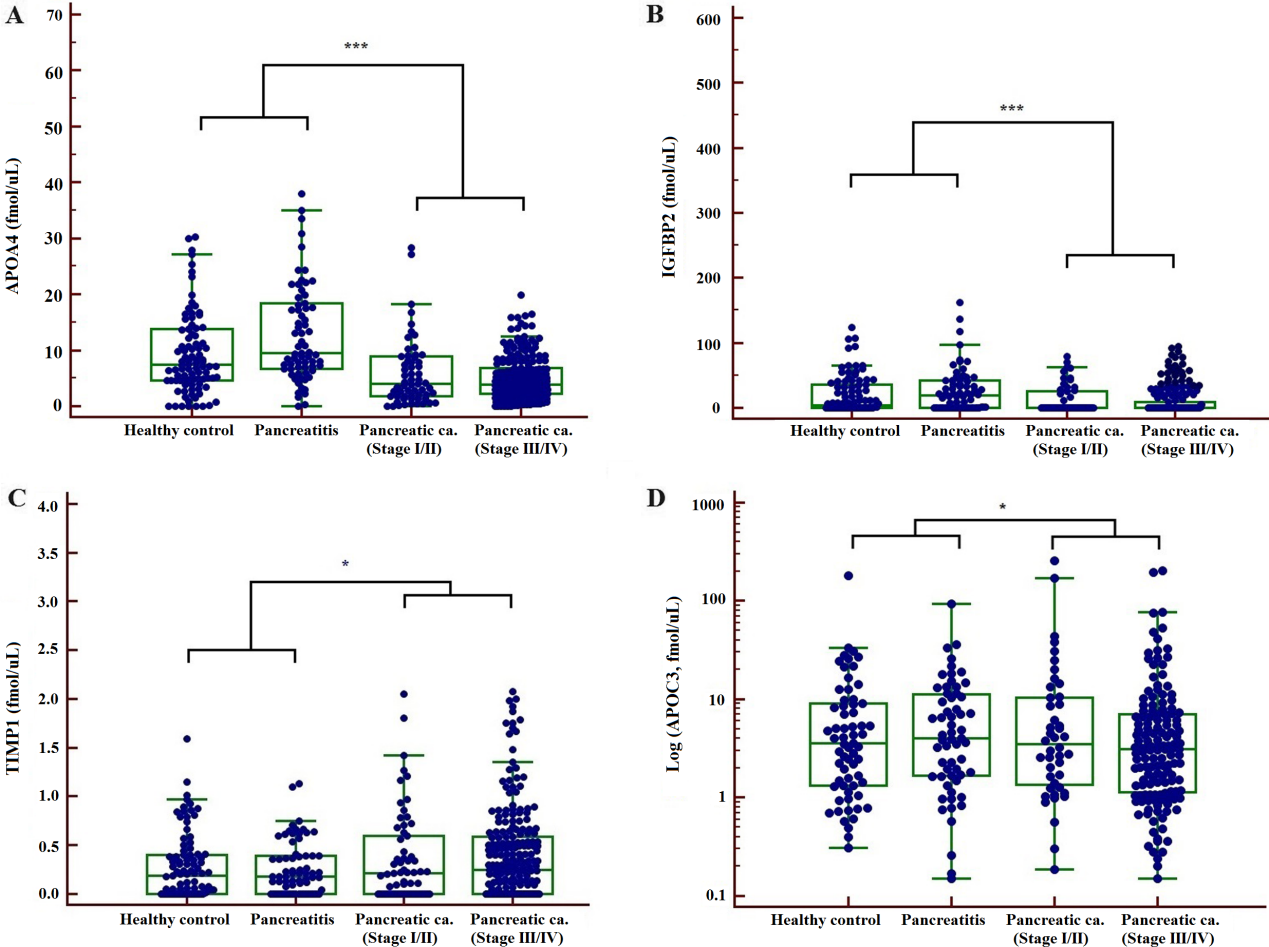
Concentrations of 4 biomarkers, APOA4 (**A**) IGFBP2 (**B**) TIMP1 (**C**) and APOC3 (**D**) measured in patient serum samples by SID-MRM-MS (*P < 0.05, ***P < 0.001).

In the validation phase the serum concentrations of these 9 proteins were measured for 456 clinical samples (94 healthy subjects, 70 pancreatitis and 292 PCs) using MS-based absolute quantification (SID-MRM-MS). Four proteins (APOA4, APOC3, IGFBP2 and TIMP1) were significantly altered in PC compared with healthy subjects or pancreatitis using Kruskal-Wallis test (*P* < 0.01) (Table 1). The higher level of TIMP1, the lower the level of APOA4, APOC3 and IGFBP2 in PC, compared with healthy controls or patients with pancreatitis (Figure 2).

We tested the diagnostic performance of each biomarker candidate or marker combination using receiver operating characteristic (ROC) analysis. Unfortunately, none of the identified proteins alone were superior to CA 19-9. Even though the sensitivity of CA 19-9 for detecting early PC remains controversial [22], CA 19-9 is still recognized as a clinically useful tumor marker. In the current study, CA 19-9 showed outstanding performance as a single marker in inter-group comparison compared with other protein markers, but interestingly showed inferior performance in distinguishing early PC from pancreatitis (sensitivity [SN] = 71% at fixed 90% specificity [SP]) than for early PC versus healthy controls (SN = 94% at fixed 90% SP).

**Figure 3:**
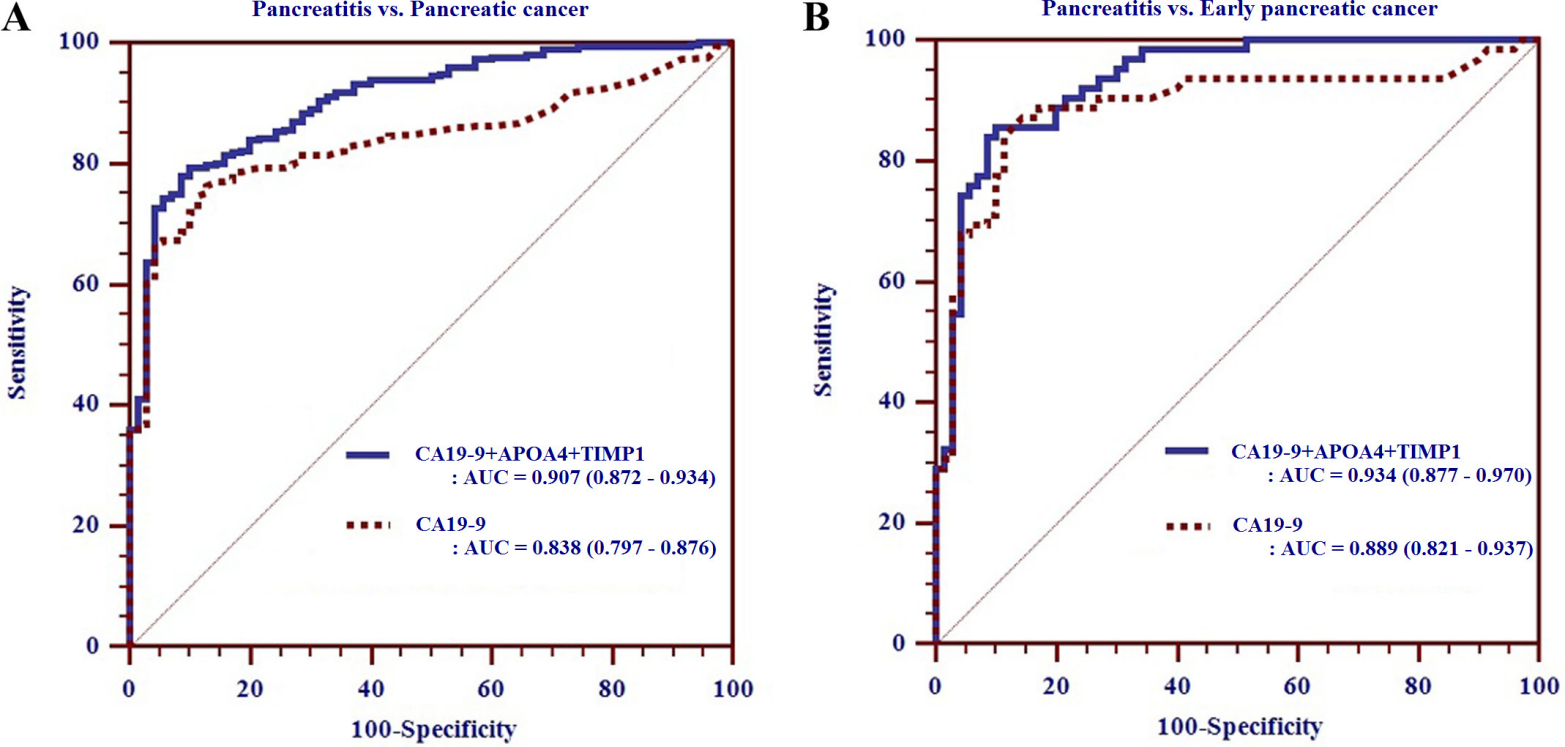
Receiver operating characteristic curve analysis of biomarker panel composed of CA19-9, APOA4 and TIMP1 for pancreatitis versus pancreatic cancer (**A**) and early pancreatic cancer (**B**) in the validation phase.

We generated biomarker panels combining CA 19-9 with the additional four biomarkers to improve the diagnostic performance for early detection of PC. Of these marker combinations, a biomarker panel consisting of CA19-9, APOA4 and TIMP 1 [AUC_panel_ > 0.934 (95% CI: 0.877–0.970), SN/SP of 86%/90%] demonstrated better performance than any other biomarker panels, or CA 19-9 alone, for distinguishing early PC from pancreatitis (Table 3, Figure 3).

**Table 3:** Diagnostic performance of single or multiple biomarkers for the detection of pancreatic cancer

Marker	% Sensitivity at fixed 90% Specificity					
	Pancreatic cancer vs.			Early pancreatic cancer vs.		
	Healthy subjects + Pancreatitis	Healthy subjects	Pancreatitis	Healthy subjects + Pancreatitis	Healthy subjects	Pancreatitis
CA19-9	79.4	84.9	69.1	88.7	93.6	71.0
APOA4	27.1	16.4	51.4	32.3	22.6	50.0
APOC3	35.3	35.3	35.3	29.0	11.3	29.0
IGFBP2	30.5	30.5	30.5	35.5	35.5	35.5
TIMP1	17.7	14.7	21.6	17.7	14.5	22.6
CA199+APOA4	73.3	84.9	73.3	29.0	12.9	75.8
CA199+APOC3	71.6	84.3	71.6	40.3	40.3	72.6
CA199+IGFBP2	71.9	84.3	71.9	8.1	9.7	77.4
CA199+TIMP1	72.6	84.3	72.6	40.8	41.4	75.8
CA199+APOA4+APOC3	81.9	82.9	74.0	21.0	14.5	74.2
CA199+APOA4+IGFBP2	80.1	81.9	79.1	29.0	12.9	80.7
CA199+APOA4+TIMP1	43.5	42.5	79.5	27.4	25.8	85.5
CA199+IGFBP2+TIMP1	78.4	81.2	74.0	16.1	16.1	74.2

Abbreviations: CA 19-9, carbohydrate antigen 19-9; APOA4, apolipoprotein A-IV; APOC3, apolipoprotein CIII; IGFBP2, insulin-like growth factor binding protein 2; TIMP1, tissue inhibitor of metalloproteinase

### Additional validation with immunohistochemistry and luminex assay

Immunohistochemical staining and Luminex-based biomarker multiplex assays were additionally performed to support our findings from the discovery phase. IHC staining of 9 proteins, including TIMP1, MUCs, and VCAN, was performed on 210 pancreatic tissue lesions and a multiplex assay for TIMP-1, -2, -3 and -4, MMP-1,-7,-8 and -9, and IGFBP-1, -2, -3 -4 and -5 was performed on 220 patient sera samples.

**Figure 4:**
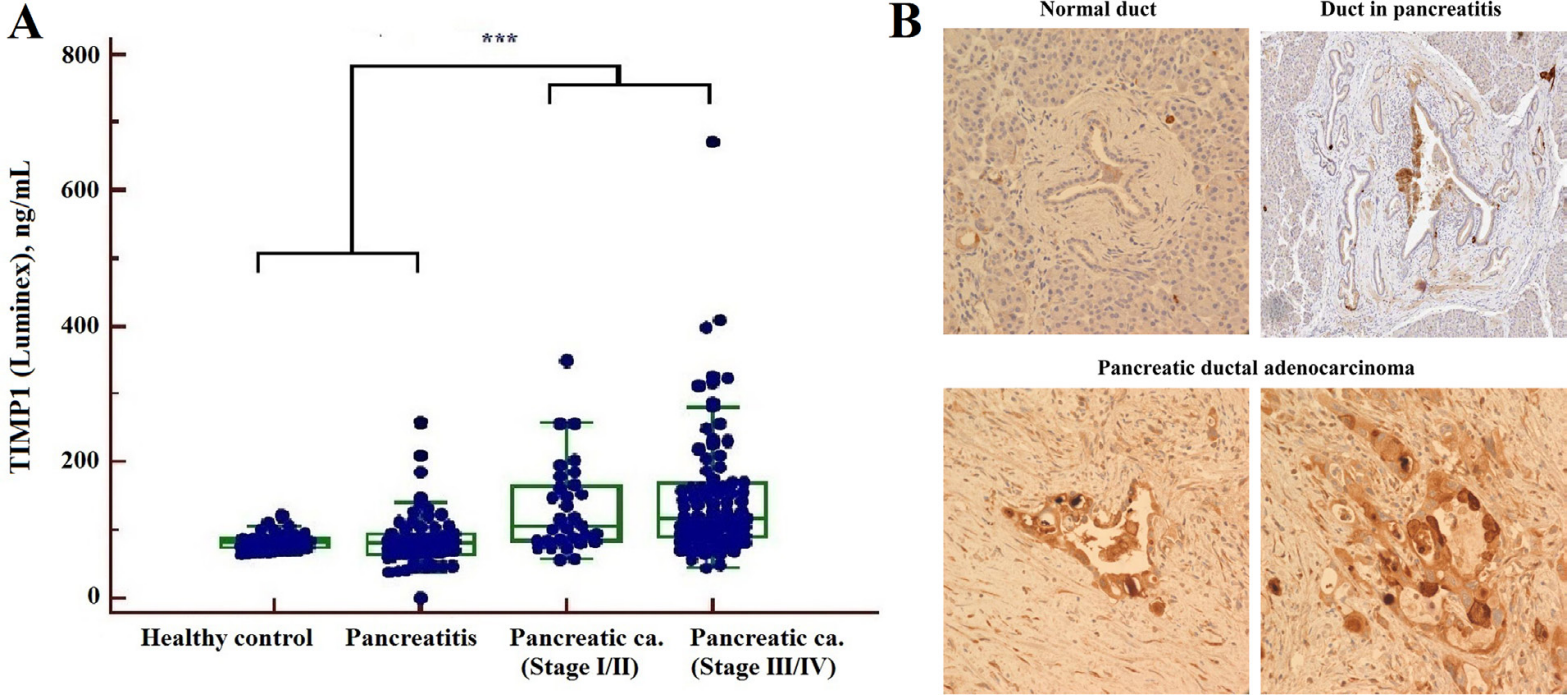
Determination of TIMP1 by Luminex assay (**A**) and immunohistochemistry staining (**B**) (***P < 0.001): The serum levels of TIMP1 were increased in PC compared with healthy controls and pancreatitis by Luminex. TIMP1 expression was absent in pancreatic duct of normal and chronic pancreatitis, but was increased in pancreatic ductal adenocarcinoma by immunohistochemistry staining.

Our present data were concordant with the results of previous studies [23–25], showing an increase in TIMP1 levels in the PC group by the Luminex assay (Figure 4) whereas levels of TIMPs (−2, -3 and -4) were not significantly altered compared with healthy subject or the pancreatitis group. None of the MMPs and IGFBPs showed statistical significance in our data acquired from MRM-MS or multiplex assay. The results of IHC staining for TIMP1 showed stronger staining for PC than for normal or pancreatitis ducts. Fifty of 70 PCs (75.7%) had positive results on IHC staining for TIMP1 (Supplementary Table 2, Figure 4).

## DISCUSSION

Early diagnosis of pancreatic cancer is ultimately important for enhancing the survival of patients; however, screening tests with sufficient sensitivity and specificity for early detection of PC in clinical practice are not currently available.

Despite its dubious effectiveness, serum CA 19-9 is the only marker approved by the FDA as a tumor marker for PC. Low sensitivity of the test has been reported for early detection of PC. We performed an integrated analysis of proteomic and transcriptomic data to develop a diagnostic platform for the detection of PC. A bioinformatics approach was helpful to mine for useful markers in a less labor intensive and time consuming but effective manner. Here, we employed MS and a bioinformatics-based approach to effectively discover and evaluate biomarker candidates for diagnosis of early PC. A large number of proteins were monitored and selected through our successfully established comprehensive screening strategy, leading to the identification of APOA4, APOC3, IGFBP2 and TIMP1, proteins associated with acute phase, growth factor or metastasis, as potential biomarker candidates.

In recent decades, the acute phase proteins have attracted attention as potential biomarkers due to the likelihood of being associated with cancer cell growth and metastasis [26–28]. The current study included several acute phase proteins, such as apolipoprotein A4 (APOA4), apolipoprotein C1 (APOC1), apolipoprotein C3 (APOC3), apolipoprotein E (APOE), α1 glycoprotein (AGP), α1 antichymotrypsin (ACT), S100 protein, and clusterin (CLU).

Previous research showed altered expression levels of MMPs and their tissue inhibitors (TIMPs), which are involved in tissue remodeling and stimulate tumor cell progression, in various cancers. TIMPs have been strongly implicated as potential markers in the diagnosis of pancreatic cancer [23, 29, 30]; in particular, TIMP1 is associated with tumor metastasis and has long been a protein of interest. In our study, serum TIMP1 levels were significantly increased in PCs compared to pancreatitis by MRM assay, IHC and multiplex immunoassay. Moreover, we previously showed that levels of serum MMPs were altered in a multiplex immunoassay [29], but unfortunately MMPs were undetectable by MRM in the current study, probably due to the limited sensitivity of the mass spectrometry-based assay.

To date, several different approaches have been applied to mine biomarkers for detection of PC. Shaw et al. [31] measured the serum levels of 27 cytokines by a multiplex immunoassay in 241 PC patients and showed that the panel of IP-10, IL-8, IL-1b and PDGF showed improved diagnosis of PC over CA19-9 alone. Brand et al. [32] analyzed 83 serum proteins using a multiplex immunoassay for the biomarker discovery of PC. A biomarker panel of CA 19-9, CEA and TIMP1 discriminated PC from benign tissue. More recently, Chan et al. [33] reported that a biomarker panel consisting of CA 19-9, CA125 and LAMC2 improves on the performance of CA 19-9 alone. Unfortunately, an effective, reliable novel biomarker that facilitates early diagnosis of PC has yet to be discovered. Together, these results indicated that none of the candidate markers had better diagnostic performance than CA 19-9 alone, but combinations of CA 19-9 and additional biomarkers showed improved results.

Thus, we generated biomarker panels composed of CA19-9 and our protein candidates and evaluated the diagnostic performances of each marker or these marker combinations in pancreatitis versus PC or Early PC. The panel composed of CA19-9, APOA4 and TIMP1 showed the best performance among the biomarker combinations to detect early PC (86% SN at 90% SP with AUC of 0.934).

In conclusion, even though none of the identified biomarker candidates alone showed better diagnostic value than CA 19-9, the biomarker panel identified in the current study showed better performance to discriminate early PC from pancreatitis than that reported in previous studies. Further studies including larger early PC populations will be needed to confirm these findings, but we expect that this panel including CA 19-9 will contribute to improved early detection of PC.

## MATERIALS AND METHODS

### Materials

Multiple affinity removal system (MARS) column and a 24-well setup and a 24 cm, pH 3–10 IPG strip were purchased from Agilent Technologies (Santa Clara, CA, United States). Sequencing grade modified trypsin was from Promega (Madison, WI, United States).

Nine isotope-labeled peptides were synthesized as internal standards (ISs) for SID-MRM-MS (AnyGen Co., Gwangju, Korea): NLSEAQL*R, TEDTIFLR, YGAATF*TR, ISASAEEL*R, GWVTDGFSSL*K, LIQGAP*TIR, AEDAPGVPL*R, VSVTL*SGK and GFQALGDAADI*R for Calmodulin-like protein 5 (CALML5), Alpha-1-acid glycoprotein 2 (ORM2), Pregnancy zone protein (PZP), Apolipoprotein A-IV (APOA4), Apolipoprotein C-III (APOC3), Insulin-like growth factor-binding protein 2 (IGFBP2), Mucin-5AC (MUC5AC), Pancreatic triacylglycerol lipase (PNLIP) and Metalloproteinase inhibitor 1 (TIMP1), respectively. * represents the amino acid labeled with the ^13^C^15^N heavy isotope.

### Study subjects

The Institutional Review Board of Samsung Medical Center (Seoul, Korea) approved this study. Peripheral blood was collected from patients with PC or pancreatitis prior to any therapeutic procedures, including surgery, chemotherapy, and radiotherapy, between June 2008 and July 2011. Enrolled patients were diagnosed based on histological and cytological findings, and imaging findings from computed tomography (CT), magnetic resonance imaging (MRI), and endoscopic ultrasound (EUS) studies. PCs were classified into four groups (stage I, stage II, stage III, and stage IV) according to tumor, node, metastasis (TNM) classification. We recruited healthy people with no clinical or biochemical evidence of pancreas-related diseases and patients with chronic or acute pancreatitis as control subjects.

We enrolled 182 specimens (116 PCs, 31 cases of pancreatitis, and 35 healthy controls) and 456 specimens (291 PCs, 70 cases of pancreatitis, and 94 healthy subjects) for the discovery and validation set, respectively. All serum specimens were obtained by centrifugation of blood at 2,330 × g for 5 min and stored at −70°C for further proteomic analysis. For additional antibody-based validation, we used pancreatic duct tissues from 70 individuals for immunohistochemical staining (IHC) and serum specimens from 220 individuals for Luminex analysis.

### Selection of biomarker surrogates

We identified PC biomarker candidates through a meta-analysis of seven PC gene expression studies [34–40]. A total of 200 microarray experiments on pancreatic ductal adenocarcinoma, pancreatic intraepithelial neoplasia, and chronic pancreatitis patient samples, in addition to normal controls, were collected from NCBI Gene Express Omnibus, EBI ArrayExpress, and Oncomine. To address cellular and genetic heterogeneity of cancer samples, we adopted a pathway-based approach where pathways rather than individual genes are considered as units of oncogenic processes. For each of seven gene expression datasets, we identified pathways whose activities were significantly altered in PC samples compared to controls (chronic pancreatitis or normal pancreas samples). For each dysregulated pathway, we extracted a subset of member genes (COndition-Responsive Genes [CORGs]) whose combined expression level represented the altered pathway activity (see ref. [41] for details). To identify robust candidate markers, we evaluated the accuracy of pathways and CORGs extracted from one study predicting disease phenotypes of the samples from the other six studies. We prioritized pathways and CORGs based on their reproducibility in independent samples and annotated whether DNA copy number changes of CORGs are consistent with their expression changes (i.e., CORGs with copy gain in PC samples are expected to show increased expression in PC). We also annotated CORGs for their cellular localization.

Mass spectrometric data were analyzed to investigate the differentially expressed proteins in PC by spectral counts. All MS/MS spectra were searched using the SEQUEST (rev.3.3.1 sp1) search engine (Thermo Fisher Scientific Inc, MA, USA) by the target decoy search strategy with the human database from the NCBI for peptide identification.

### Sample preparation and MS-based experiments

To reduce the complexity, MARS (Agilent Technologies, Santa Clara, CA, USA) was used to remove the top six most abundant proteins in serum (i.e., albumin, transferrin, IgF, IgA, anti-trypsin, and haptoglobin) according to the manufacturer's recommendations. Depleted serum samples (300 g) were subjected to tryptic digestion and then pI-based peptide separation was achieved using Agilent 3100 OFFGEL Fractionator (Agilent Technologies, Santa Clara, CA, USA) according to the manufacturer's instructions.

The tryptic peptides were analyzed in a LTQ ion trap mass spectrometer (Finnigan, CA, USA) equipped with a nano-electrospray ion source for global proteome identification. Levels of proteins of interest were determined using a QTRAP 5500 (ABSciex, Framingham, MA, United States) in MRM mode as following parameter: spray voltages, 2.2~2.5 kV range; curtain gas and spray gas; 20 and 20, respectively; the collision gas, high; The declustering potential (DP), 100 V. Mass resolution was set to units in advanced MS parameter. The tryptic peptides were loaded onto nano cHiPLC columns (75 μm × 15 cm ChromXP C18-CL 3 μm 300 Å) and separated by the Eksigent nanoLC-1D plus system combined with the cHiPLC-nanoflex system. An elution gradient of 3–55% buffer B for 30 min followed by 40–95% buffer B over 10 min were used at a flow rate of 300 nl/min.

A total of 2,292 MRM transitions against 90 proteins were subjected to the MRM experiment optimization process. Collision energy for the target peptides was automatically calculated by in silico tryptic digestion. MRM transitions with signal to noise ratio (S/N) greater than 8 were chosen through 5 replicates of the peptide mixture (data not shown). We finally selected the specific peptides and their best MRM transitions, which is used quantitative ion pairs to detect 9 proteins for the clinical validation (Supplementary Table 3).

### Validation of biomarker candidates by Luminex technologies and immunohistochemistry

The serum levels of metallopeptidase inhibitors (TIMPs [TIMP-1, -3 and -4]), matrix metalloproteinases (MMPs [MMP-1, -7, -8 and -9]) and insulin-like growth factor binding proteins (IGFBPs [IGFBP-1, -2, -3, -4 and -5]) were determined using Fluorokine MAP multiplex kits (R&D Systems, Minneapolis, MN, USA) and read on a Bioplex Luminex analyzer (Bio-Rad Laboratories, Hercules, CA, USA) as previously described [29].

Tissue microarrays (TMAs) were prepared from archival formalin-fixed paraffin-embedded sections of normal pancreatic ductal cells, ductal cells of chronic pancreatitis and invasive ductal adenocarcinomas (70 samples of each). For TMA construction, representative portions containing histologically defined normal pancreas, chronic pancreatitis and ductal adenocarcinomas were circled on the glass slides and used as a template. A 1-mm core was punched from the donor block to ensure that adequate tissue would be incorporated in the spot. Unstained 5-μm sections were cut from each TMA block and deparaffinized using routine techniques before soaking in 200 mL of Target Retrieval Solution (pH 6.0, Envision Plus Detection Kit; Dako, Carpenteria, CA, USA) for 20 min at 100°C. After cooling for 20 min, the slides were quenched with 3% H_2_O_2_ for 5 min before incubation with monoclonal antibodies against MUC1, MUC4, MUC5AC, TIMP1, ERBB2, VCAN, NOTCH3, ATP5D, and SERPINB5. IHC staining was performed with an automated immunostainer (Bond-max, Leica). The protocol is summarized in Supplementary Table 4. Labeling was detected with the Dako Envision system according to the manufacturer's protocol. All sections were counterstained with hematoxylin. IHC staining results were scored by one pathologist based on the intensity and the percentage of positive cells as follows: 0, absent; 1, focal (< 10%); 2, partial (10–50%); and 3, diffuse (> 50%). IHC stain results were classified according to semi-quantitative score as negative (score 0 or 1) or positive (score 2 or 3).

### Statistical analysis

Data were analyzed using the SPSS statistical package ver. 21.0 (IBM Corporation, Somers, NY, USA), and MedCalc software ver. 16.8.4 (Mariakerke, Belgium). Differences in the concentration of markers between groups were assessed using the Kruskal-Wallis test.

Pearson`s Chi-square (χ2) or Spearman`s rank correlation coefficient test was used to assess possible relationships between various parameters. A *P-value* ≤ 0.05 was considered statistically significant. ROC analysis was used to assess the diagnostic performance of proteomic markers and the area under the curve (AUC) was estimated.

## SUPPLEMENTARY TABLES




