# Supplementary material for: Large-scale clinical validation of biomarkers for pancreatic cancer using a mass spectrometry-based proteomics approach
Source: Oncotarget. 2017 Apr 27;8(26):42761–71. doi: 10.18632/oncotarget.17463 (PMC5522104; doi:10.18632/oncotarget.17463)
Supplement: Supplementary file 1 [file oncotarget-08-42761-s001.pdf]

Large-scale clinical validation of biomarkers for pancreatic cancer using a mass spectrometry-based proteomics approach

Supplementary Materials

Supplementary Table 1: Proteins identified using pathway-based gene expression meta-analysis and shotgun proteomics in the discovery phase. See Supplementry_Table_1

Supplementary Table 2: Immunohistochemistry staining results of 9 proteins

	Positive case (%)		
**Gene Symbol**	**Normal duct**	**Duct in Pancreatitis**	**Ductal Adenocarcinoma**
MUC1	0% (0/70)	27.1% (19/70)	64.3% (45/70)
MUC4	0% (0/70)	0% (0/70)	61.4% (43/70)
MUC5AC	0% (0/70)	21.4% (15/70)	42.8% (30/70)
SERPINB5	0% (0/70)	17.1% (12/70)	31.4% (22/70)
ERBB2	0% (0/70)	15.7% (11/70)	12.9% (9/70)
VCAN	0% (0/70)	7.14% (5/70)	51.4% (36/70)
NOTCH3	0% (0/70)	31.4% (22/70)	61.4% (43/70)
ATP5D	14.3% (10/70)	35.7% (25/70)	64.3% (45/70)
TIMP1	0% (0/70)	2.3% (2/70)	75.7% (53/70)

Supplementary Table 3: MRM transitions for 9 proteins

Marker	Q1	Q3	dwell time (msec)	peptide sequence	Target ion		Collision energy (eV)
Alpha-1-acid glycoprotein 2	497.76	764.43	40	TEDTIFLR	+2y6		26.8
	497.76	649.40	40		+2y5		26.8
	501.27	771.45	40	TEDTIFL*R	+2y6	IS	26.8
	501.27	656.42	40		+2y5		26.8
Apolipoprotein A-IV	488.26	775.39	40	ISASAEELR	+2y7		26.4
	488.26	704.36	40		+2y6		26.4
	491.77	782.41	40	ISASAEEL*R	+2y7	IS	26.4
	491.77	711.37	40		+2y6		26.4
Apolipoprotein CIII	598.80	953.49	40	GWVTDGFSSLK	+2y9		30.4
	598.80	854.43	40		+2y8		30.4
	602.31	960.51	40	GWVTDGFSSL*K	+2y9	IS	30.4
	602.31	861.44	40		+2y8		30.4
Insulin-like growth factor binding protein 2	484.80	742.42	40	LIQGAPTIR	+2y7		26.3
	484.80	614.36	40		+2y6		26.3
	487.80	748.43	40	LIQGAP*TIR	+2y7	IS	26.3
	487.80	620.38	40		+2y6		26.3
Pancreatic triacylglycerol lipase	395.74	604.37	40	VSVTLSGK	+2y6		23.1
	395.74	505.30	40		+2y5		23.1
	399.25	611.38	40	VSVTL*SGK	+2y6	IS	23.1
	399.25	512.32	40		+2y5		23.1
Pregnancy zone protein	443.72	723.38	40	YGAATFTR	+2y7		24.8
	443.72	595.32	40		+2y5		24.8
	448.74	733.41	40	YGAATF*TR	+2y7	IS	24.8
	448.74	605.35	40		+2y5		24.8
Inhibitor of metalloproteinase 1	617.31	830.44	40	GFQALGDAADIR	+2y8		31.1
	617.31	717.35	40		+2y7		31.1
	620.82	837.45	40	GFQALGDAADI*R	+2y8	IS	31.1
	620.82	724.37	40		+2y7		31.1
Mucin 5AC glycoprotein	512.77	709.44	40	AEDAPGVPLR	+2y7		27.3
	512.77	638.40	40		+2y6		27.3
	516.28	716.45	40	AEDAPGVPL*R	+2y7	IS	27.3
	516.28	645.42	40		+2y6		27.3
Calmodulin-like protein 5	465.75	703.37	40	NLSEAQLR	+2y6		25.6
	465.75	616.34	40		+2y5		25.6
	469.26	710.39	40	NLSEAQL*R	+2y6	IS	25.6
	469.26	623.36	40		+2y5		25.6

Supplementary Table 4: Antibodies used in this study

Antibody	Incubation time	Dilution	Supplier
MUC1	20 min	1:200	Novocastra
MUC4	20 min	1:100	Santa Cruz
MUC5AC	20 min	1:200	Novocastra
TIMP1	20 min	1:200	DAKO
ERBB2	20 min	1:300	Novocastra
VCAN	20 min	1:50	Novus
NOTCH3	20 min	1:150	Novus
ATP5D	20 min	1:150	Sigma
SERPINB5	90 min	1:100	BD Biosciences
